# Cell‐blocks are suitable material for programmed cell death ligand‐1 immunohistochemistry: Comparison of cell‐blocks and matched surgical resection specimens in lung cancer

**DOI:** 10.1111/cyt.12743

**Published:** 2019-07-19

**Authors:** Min Gyoung Pak, Mee Sook Roh

**Affiliations:** ^1^ Department of Pathology Dong‐A University College of Medicine Seo‐gu Busan South Korea

**Keywords:** cell‐blocks, cytology, immune checkpoint therapy, lung cancer, matched surgical resection specimens, programmed cell death ligand‐1

## Abstract

**Objective:**

Programmed cell death ligand‐1 (PD‐L1) has emerged as a predictive biomarker in lung cancer. PD‐L1 immunohistochemistry (IHC) assay predicts the response to immunotherapy, but cytology specimens are often the only samples available in a considerable proportion of advanced lung cancer patients. We delineate practical feasibility and efficacy of cytology cell‐block (CB) specimens for PD‐L1 expression and concordance between cytology CBs and surgical resection specimens.

**Methods:**

In total, 58 eligible patients with primary lung cancer who received computed tomography‐guided percutaneous needle aspiration and surgery were included. PD‐L1 IHC (clone SP263) was performed on CBs prepared from residual liquid‐based cytology material and matched surgical resection specimens. PD‐L1 positive tumour cell proportion was categorised in four score groups: (a) <1%; (b) ≤1% to <10%; (c) ≤10% to <50%, (d) ≥50%.

**Results:**

Comparison of PD‐L1 expression in cytology CBs and matched surgical resection specimens showed a high concordance (κ value 0.65). According to the therapeutic guideline of immunotherapeutic agents, a positive percent agreement was 94.34%, and a negative percent agreement was 100% at a cut‐off value for positivity of 1% PD‐L1 expression. There was a significant difference observed with regard to rates of PD‐L1 positivity when comparing smoking history (*P = *0.02), age (*P = *0.04) and pathological TNM stage (*P = *0.04).

**Conclusions:**

The results show that cytology CBs evaluated for PD‐L1 IHC assay have high concordance with matched surgical resection specimens and can be used for assessing PD‐L1 expression. Also, we propose that CBs are suitable materials for evaluating PD‐L1 expression while simultaneously performing both diagnostic and molecular tests.

## Abstract 2

This study showed that cytology cell‐blocks (CBs) evaluated for PD‐L1 immunohistochemistry (IHC) assay have high concordance with matched surgical resection specimens and can be used for assessing PD‐L1 expression. Also, we propose that CBs are suitable materials for evaluating PD‐L1 expression while simultaneously performing both diagnostic and molecular tests.

## 1 INTRODUCTION

Despite recent advances in molecular targeted therapies, lung cancer is the most common cause of cancer‐related death.^1^ The 5‐year survival rates of lung cancer patients were reported as 4%‐17% according to tumour stage and location.^2^ Despite an initial dramatic response, almost all non‐small cell lung cancer (NSCLC) patients harbouring an epidermal growth factor receptor (EGFR) mutation eventually showed disease progression due to acquired resistance to EGFR tyrosine kinase inhibitor treatment within 1 year.^3^ In the absence of activating mutation of EGFR or translocation/gene rearrangements of anaplastic lymphoma kinase and ROS proto‐oncogene 1, platinum‐based chemotherapy remains the first‐line treatment for advanced stage NSCLC.^4^

It seems that ceaseless effort to overcome the limitation of conventional cytotoxic agents and target therapies may finally bear fruit. Recently, many clinical trials using immunotherapeutic agents, particularly immune checkpoint inhibitors, have shown signs of improvement.^2^, ^4^, ^5^, ^6^, ^7^, ^8^ Programmed cell death ligand‐1 (PD‐L1) expression is a predictive biomarker for anti‐PD‐L1 immunotherapy in lung cancer.^9^ Clinical trials with anti‐PD‐1 immune checkpoint inhibitors have shown an association between the magnitude of clinical efficacy and the level of PD‐L1‐expression as evaluated by PD‐L1 immunohistochemistry (IHC).^4^, ^6^, ^7^, ^10^, ^11^, ^12^ PD‐L1 IHC assay is a suitable diagnostic method for precise targeted therapy and is an easily performed, relatively inexpensive, and widely accessible method. According to recent reports, anti‐PD‐1 immune checkpoint inhibitors including pembrolizumab, nivolumab, atezolizumab and durvalumab resulted in significantly longer overall survival for patients with advanced NSLCL.^4^, ^5^, ^6^, ^7^, ^8^, ^10^ However, it is often difficult to obtain tumour tissue in patients with advanced NSLCL because of tumour location and patient comorbidity.^13^ Therefore, diagnostic lung cancer samples are often very small biopsy tissues or cytology samples. In a considerable proportion of lung cancer patients, cytology specimens are the only samples available for diagnosis. In this setting, PD‐L1 IHC on cytology specimens has become critical. Expert consensus is that cytology specimens are suitable not only for diagnostic purposes but also for molecular testing.^14^, ^15^ However, the use of cytology specimens for assessing PD‐L1 expression is not advocated, because little is validated for this purpose.

In this study, we delineate the practical feasibility and efficacy of cytology cell‐block (CB) specimens for PD‐L1 expression and concordance between cytology CB and surgical resection specimens. Finally, we would like to determine whether the PD‐L1 IHC on cytology CBs can be a suitable method for making future immunotherapy treatment decisions.

## 2 MATERIALS AND METHODS

### 2.1 Patients and samples

After institutional review board approval was obtained, a retrospective review of pathology reports was done. A total of 58 patients underwent computed tomography (CT)‐guided percutaneous needle aspiration (PCNA) and surgical resection from December 2011 to June 2017 at Dong‐A University Medical Center, Busan, South Korea. Eligible paired samples from primary lung cancer without preoperative chemotherapy or radiotherapy history were obtained. Excluded were cytology CB specimens containing fewer than 100 viable tumour cells.

All pathological slides were reviewed independently by two pathologists, and the histological type, pleural invasion, lymph node metastasis, lymphatic invasion and vascular invasion were re‐evaluated. Diagnoses were made according to the 2015 World Health Organisation classification of lung tumours and stages were determined according to the 7th edition of the American Joint Committee on Cancer TNM staging system.^16^, ^17^ Other clinicopathological data including age, sex, smoking history, distant metastasis, EGFR mutation, KRAS mutation, anaplastic lymphoma kinase rearrangement and survival data were obtained from pathological and medical records. Follow‐up data were included up to June 2018 or until death or loss of follow‐up.

This study was approved by the Institutional Review Board (IRB) at Dong‐A University Medical Center (DAUHIRB‐18‐009). Written informed consent was obtained from all of the patients. All specimens were handled and made anonymous according to the ethical and legal standards.

### 2.2 Cell‐block preparation

All cytology specimens were obtained by CT‐guided PCNA and fixed with CytoRich™ Red (Thermo Fisher Scientific). Each sample was processed for both one liquid‐based cytology (LBC) and one CB slide. LBC slides were prepared using SurePath™ (Thermo Fisher Scientific, Becton, Dickinson and Co.) according to the manufacturer's instructions and were stained with Papanicolaou stain. The residual SurePath™ samples for CB slides were immediately centrifuged at 689g for 5 minutes and embedded into paraffin after fixation in 10% buffered neutral formalin for 6 hours at room temperature. Tissue processing was conducted by Leica PELORIS II Rapid Tissue Processor (Leica Microsystems). CBs were cut into 4‐μm thick sections and stained with haematoxylin and eosin using the standard method.

### 2.3 PD‐L1 IHC

PD‐L1 IHC using SP263 anti‐PD‐L1 clone (Ventana Medical Systems) was stained by a VENTANA benchmark ULTRA platform optimised with the OptiView DAB IHC Detection kit (Ventana Medical Systems) according to the manufacturer's instructions. Sections of placenta were included as positive controls and negative controls ran concurrently for each case.

All pathological slides were reviewed independently by two pulmonary pathologists. If there was a discrepancy in independent opinions, the slides were reviewed together to achieve a consensus. The consensus judgments were adopted as the final results. The scoring was performed in a blinded fashion for each other and for the relationship between cytology and histology pairs. Viable tumour cells exhibiting partial or complete membranous staining with any staining intensity were considered as positive. Cytoplasmic staining without any membranous staining was excluded. PD‐L1 expression was categorised in four score groups according to the following cut‐offs:

1. score 0: no staining cells or less than 1% of tumour cells;
2. score 1: at least 1% of tumour cells but less than 10%;
3. score 2: at least 10% of tumour cells but less than 50%;
4. score 3: more than 50% of tumour cells.

### 2.4 Statistical analysis

Statistical analyses were conducted using the statistical software SPSS 22.0 for Windows (SPSS Inc.). The κ coefficient of concordance was calculated, and the agreement was classified as follows (a) weak: κ values within 0.21‐0.40; (b) moderate: κ values within 0.41‐0.60; (c) good: κ values within 0.61‐0.99. The χ^2^ test was used to assess PD‐L1 expression with respect to clinicopathological parameters. The survival curves of the patients were determined using the Kaplan‐Meier method, and the log‐rank test was used for statistical evaluations. A probability (*P*)‐value ≤0.05 was considered statistically significant.

## 3 RESULTS

### 3.1 Clinical and pathological characteristics

This study included 58 primary lung patients representing 35 men and 23 women. The median age at diagnosis was 66 years (range, 33‐84 years). Histologically, 38 tumours (65.52%) were classified as adenocarcinoma, 16 (27.59%) as squamous cell carcinoma, two (3.45%) as adenosquamous carcinoma and two (3.45%) as small cell carcinoma. Pathological T stage consisted of pT1 for 19 (32.76%), pT2 for 32 (55.17%) and pT3 for seven (12.07%). Twenty‐eight of the patients (48.28%) had pleural invasion and 24 (41.38%) had lymph node metastasis. The univariate analysis using the χ^2^ test revealed that PD‐L1 expression was significantly correlated with smoking history (*P = *0.02), age (*P = *0.04) and pathological TNM stage (*P = *0.04). Details of clinical and pathological factors are shown in Table 1.

**Table 1:** Relationship between PD‐L1 expression rates and clinicopathological factors

Clinicopathological factors	Number	PD‐L1 expression	
		Cytology cell‐block	Surgical resection
Sex			
Male	35	0.28	0.20
Female	23		
Age (y)			
≤60	17	0.07	**0.04**
>60	41		
Mean age (range)	66 (33‐84)		
Smoking history			
Never	24	**0.02**	0.07
Former	15		
Current	19		
Pack‐years (range) a	40.7 (1.7‐120)		
Histological type			
Adenocarcinoma	38		
Squamous cell carcinoma, keratinising type	10		
Squamous cell carcinoma, non‐keratinising type	6	0.70	0.35
Small cell carcinoma	2		
Adenosquamous carcinoma	2		
Pathological T stage			
pT1a	2		
pT1b	17		
pT2a	28	0.48	0.09
pT2b	4		
pT3	7		
Pathological N stage			
pN0	34		
pN1	9	0.09	0.78
pN2	15		
pN3	0		
Distant metastasis			
No	58	N/A	
Yes	0		
Pathological TNM stage			
IA	13		
IB	17		
IIA	7	0.16	**0.04**
IIB	5		
IIIA	16		
Pleural invasion			
PL0	30		
PL1	22	0.294	0.489
PL2	3		
PL3	3		
Lymphatic invasion			
No	41	0.05	0.86
Yes	17		
Vascular invasion			
No	52	0.61	0.39
Yes	6		
*EGFR* mutation			
Mutant	11	0.88	0.84
Wild type	47		
*KRAS* mutation			
Mutant	0	N/A	
Wild type	4		
N/A	54		
*ALK* rearrangement			
Mutant	0	N/A	
Wild type	18		
N/A	40		

Abbreviation: N/A, not applicable.

a Among former and current smokers only.

The median follow‐up after surgery was 28.6 months (range, 2‐61 months). Cancer‐related deaths occurred in two patients (3.45%), and one patient died of an unrelated cause. Kaplan‐Meier survival analysis with log‐rank test showed no statistically significant difference according to the expression rates of PD‐L1.

### 3.2 PD‐L1 expression in lung cancer specimens

In cytology CB specimens, eight cases (13.79%) demonstrated less than 1% expression (score 0), 27 (46.55%) were found to have 1% to 9% expression (score 1), nine (15.52%) showed 10% to 49% expression (score 2) and 14 (24.14%) demonstrated more than 50% expression (score 3). In matched surgical resection specimens, five cases (8.62%) had less than 1% positive tumour cells (score 0), 23 (39.66%) had 1% to 9% positive tumour cells (score 1), 13 (22.41%) had 10% to 49% positive tumour cells (score 2), and 17 (29.31%) had more than 50% positive tumour cells (score 3). Details are shown in Table 2.

**Table 2:** Comparison of programmed cell death ligand‐1 expression between cytology cell blocks and surgical resection specimens

	Surgical resection					Kappa value
	0	1	2	3	Total	
Cytology cell‐block						
0	5	2^a^	1^a^	0	8	0.65
1	0	21	6^a^	0	27	
2	0	0	5	4^a^	9	
3	0	0	1^a^	13	14	
Total	5	23	13	17	58	

a Discordant cases.

### 3.3 Comparison of PD‐L1 expression between cytology CBs and matched surgical resection specimens

**Figure 1:**
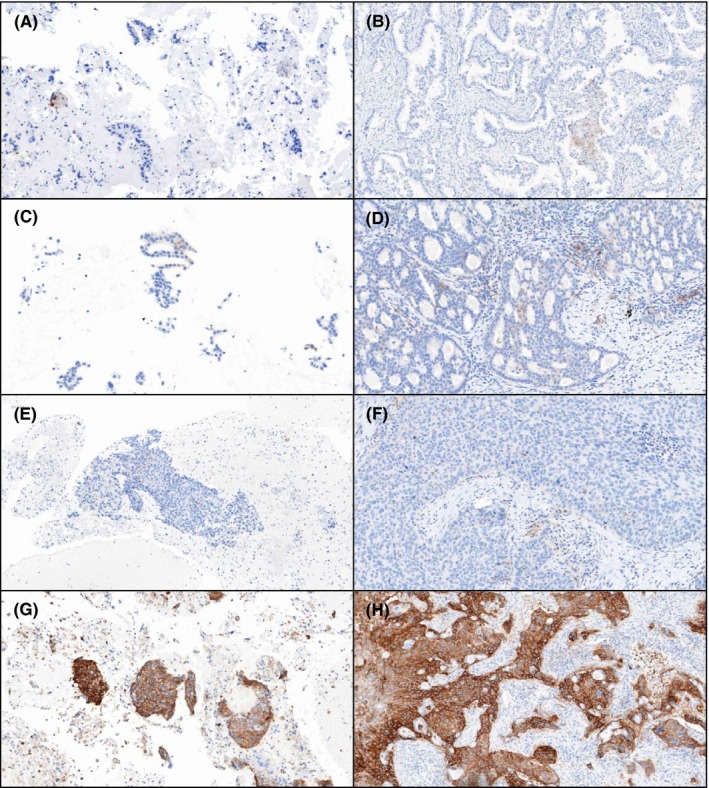
Programmed cell death ligand‐1 (PD‐L1) immunohistochemical staining on cytology cell blocks and matched histology slides (A and B) Case 1. The cytology cell block (A) and matched histology (B) slides showed no PD‐L1 positive tumour cells (×100; C and D) Case 2. Both slides showed more than 1%, but less than 10% PD‐L1 positivity in adenocarcinoma. (×100; E and F) Case 3. Squamous cell carcinoma showed more than 1%, but less than 10% PD‐L1 positivity in cytology and histology slides (×100; G and H) Case 4. High PD‐L1 expression, more than 50%, was observed in G and H (G: ×100, H: ×40)

**Figure 2:**
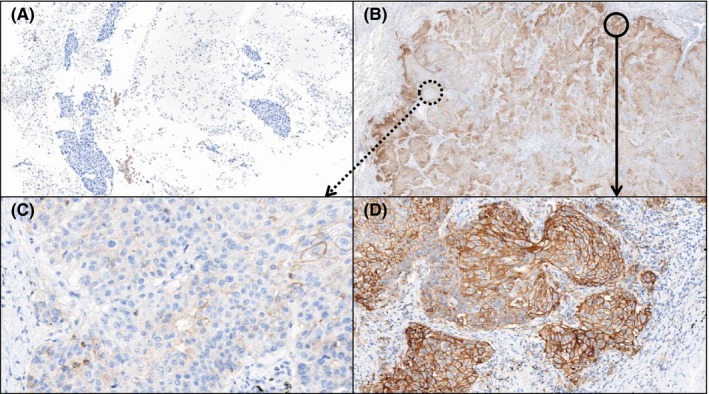
The only case of substantial PD‐L1 discrepancy (A) The cytology CB specimen showed less than 1% PD‐L1 positivity. (x40) (B) The matched surgical resection specimen from the same patient showed heterogeneous PD‐L1 positivity. The final score was 2. (x10) (C) The high magnification view of dashed line circle in Figure 2(B) showed less than 1% PD‐L1 positivity, the same PD‐L1 result of the cytology CB specimen. (x100) (D) The high magnification view of solid line circle in Figure 2(B) showed near 80% PD‐L1 positivity, the discordant PD‐L1 result of the cytology CB specimen. (x100)

All cytology samples were obtained from the same anatomical site before surgical resection. The median time interval between two specimens was 42 days (range, 7‐93 days). All patients received no treatment during the interval. Comparison of PD‐L1 expression in cytology CBs and matched surgical resection specimens showed high concordance (κ value 0.65, Table 2). Examples of PD‐L1 expression in cytology CBs and matched histology slides are shown in Figure 1. When comparing PD‐L1 expression rates in cytology CBs and matched surgical resection specimens according to the therapeutic guidelines of immunotherapeutic agents, a positive percent agreement was 94.34% and a negative percent agreement was 100% at a cut‐off value for positivity of 1% PD‐L1 expression^18^ (Table 3). All CBs that were scored as positive (score 1‐3) showed positive PD‐L1 expression in matched resection specimens without exception. Therefore, patients with positive PD‐L1 expression in cytology specimens do not need to have repeat tests using resection specimens. As a result, the time required for the treatment decision and the cost for repeat tests can be saved. Only three out of 58 cases (5.17%) showed discordant scores. These incongruous pairs showed negative results of cytology CBs, but scored 1, 1 and 2 in matched resection specimens. An example of one of the mismatch cases is shown in Figure 2.

**Table 3:** Comparison of programmed cell death ligand‐1 expression at 1% cut‐off value

	Surgical resection	
	<1%	≥1%
Cytology cell‐block		
<1%	5	3^a^
≥1%	0	50

a Discordant cases.

## 4 DISCUSSION

The neoplastic process including tumour initiation, proliferation and migration of tumour cells depends not only on the evolving genomics and molecular properties of the tumours but also on their interaction with the tumour microenvironment, which is composed of immune cells, tumour cells, stromal cells and the extracellular matrix.^2^, ^19^ Recent studies have revealed that the interaction between tumour cells, and the immune system is especially worthy of notice.^12^, ^19^ PD‐L1 on either tumour cells or host immune cells play a key role in inhibiting anti‐tumour immunity by the modification of cytotoxicity of effector T cells and the secretion of cytokines such as interferon‐γ.^20^ Currently, it is mandatory to evaluate PD‐L1 expression by IHC for the administration as anti‐PD‐1 checkpoint inhibitors in lung cancer patients.^4^, ^5^, ^6^, ^7^, ^8^

### 4.1 Comparison of PD‐L1 expression

Objective assessment of PD‐L1 expression has three major theoretical and practical issues; inter‐assay variability because of different antibody affinities, tumour heterogeneity within the tumour, and different results that depend on the timing of the biopsy.

First, although inter‐assay variability that results from different PD‐L1 antibody affinities is known,^21^, ^22^ there is substantial progress in the technical standardisation to assess PD‐L1 using IHC. In 2015, the US Food and Drug Administration approved 22‐C3 and 28‐8 IHC assays for NSCLC. Now, it is acceptable for PD‐L1 immunostaining with PD‐L1 antibodies 22‐C3, 28‐8, SP142, and SP263 on formalin‐fixed paraffin‐embedded samples.^23^, ^24^, ^25^ However, Capizzi et al reported that the accuracy of the antibody SP263 was ,higher than that of other antibodies on formalin‐fixed paraffin‐embedded and cytology samples.^22^ The PD‐L1 expression of tumour cells within cytology specimens was found to be consistent with the PD‐L1 expression of matching histology specimens, but the PD‐L1 expression of immune cells within cytology specimens cannot replace results in matched histology specimens.^26^ Here we investigated concordance rates of PD‐L1 expression in tumour cells between cytology CBs and matched surgical resection specimens using an SP263 assay, based on findings of previous reports.^21^, ^22^, ^23^, ^24^, ^25^, ^26^

Second, recent studies reported cytology‐histology correlation in lung cancer, but we have doubts about the rigorous control of heterogeneity in tumours.^25^, ^26^, ^27^, ^28^, ^29^, ^30^, ^31^ These studies were done using samples collected by various methods. Cytology specimens were collected by fine needle aspiration, bronchial washing and bronchial brushing. Surgical specimens were obtained by endobronchial biopsy, core needle biopsy, wedge resection or lobectomy. This study included only cytology cases derived from CT‐guided PCNA and processed by the same method. Surgical specimens were all lobectomies. Samples from CT‐guided PCNA have fewer normal cells than do trans‐bronchial processes, so they provide an easier way to recognise tumour cells and PD‐L1expression despite CB preparation. Although the tumour sections from lobectomies showed no even PD‐L1 expression because of tumour heterogeneity, PD‐L1 expression can be measured more accurately than from small biopsy specimens. Previous studies included comparative samples from different anatomical sites although they used paired cytology and histology specimens. Although various sampling methods have been used, substantial agreement was achieved between matched CBs and surgical specimens when CBs contain more than 100 tumour cells.^32^ However, we used matched cytology and histology specimens from the same anatomical sites. Because the purpose of this study is to compare PD‐L1 expression between cytology and histology samples and replace PD‐L1 test on resection specimens with cytology, it is more accurate to compare PD‐L1 expression from the same anatomical site. It was reported that PD‐L1 expression was different between primary lung cancer and metastatic tumours, mostly maintained, but sometimes showed positive and negative conversion at metastatic lesions.^33^ For that reason, it is necessary to investigate agreement of PD‐L1 expression between the primary tumours and metastatic lesions. Moreover, as utility of cytology specimens become more critical in advanced cancer patients, further studies are required to determine whether the PD‐L1 IHC results vary according to the cytology sampling methods. It might be helpful for predicting the therapeutic effect of PD‐1 axis therapies.

This study showed a high concordance of PD‐L1 expression between cytology CBs and matched surgical resection specimens. We assume that the concordance results from the concept of this experiment, of using comparative samples obtained from the same anatomic site and by the same method. As shown in Table 2, 44 cases out of 58 revealed almost identical positive percentages between the two slides. Among 14 discordant cases, the PD‐L1 expression rates in the histology specimens were higher than those of the CBs in 13 cases. Only one case showed a score of 3 in the cytology specimen and a score of 2 in the surgical specimen. According to the Food and Drug Administration‐approved cut‐off point of greater than 1%,^23^ only three discrepant cases remain. Two of the three showed a score of 0 in cytology CBs, whereas 3% PD‐L1 positivity (score 1) in matched resection specimens. The time interval between aspiration biopsy and lobectomy was similar to other cases, therefore it is not considered to be a bias. It is reasonable to assume that the discrepancy may result from the tumour with a very low proportion of PD‐L1 positive cells. The last one was the only case showing a substantial discrepancy, no PD‐L1 expression (score 0) in the cytology CB vs 40% (score 2) in the matched histology specimen. The histology slide showed mostly PD‐L1 negative tumour cells, but near 50% PD‐L1 positive tumour areas were also seen (Figure 2). We presumed that the cytology specimen happened to be obtained from the region with negative PD‐L1 expression of the tumour with severe heterogeneity. Although there are a few exceptions of poor cyto‐histology concordance, tumours with low rates of PD‐L1 positive cells or severe heterogeneity, we provide early evidence that cytology CBs can be successfully used for PD‐L1 immunohistochemical detection at least using the cut‐off of 1%. Furthermore, the recent study revealed that cytology CB samples showed slightly higher sensitivity for PD‐L1 immunohistochemical staining on tumour cells as compared to surgical specimens, the utility of CBs on PD‐L1 IHC is expected to increase in the future.^34^

The third potential issue is different PD‐L1 expressions depending on the timing of the biopsy. Indeed, PD‐L1 expression seems to be a dynamic biomarker. It is theoretically reasonable to change the expression rates depending on time. However, there was no change of PD‐L1 expression, at least within the interval suggested in this study. To change immunoreactivity of PD‐L1 has yielded conflicting results,^25^, ^31^ therefore the exact nature of the expression pattern of PD‐L1 varies with the timing of the biopsy is still uncertain. Further prospective studies paired with clinical outcome should be performed.

### 4.2 Clinicopathological parameters

This study indicated that PD‐L1 expression has a positive correlation with smoking history in cytology and a positive tendency in surgical resection. It is consistent with previous findings. Smoking might skew host immune response to promote lung cancer, and consequently influence the tumour microenvironment and the prognosis in NSCLC patients.^35^, ^36^ PD‐L1 expression was associated with smoking status, current smokers with more pack‐years showed higher expression rates and intensity than did never smokers.^37^ Rizvi et al have reported that the clinical efficacy of pembrolizumab correlates with a molecular signature characteristic of tobacco carcinogen‐related mutagenesis in NSCLC patients.^38^ There is no statistical significance between overall survival and PD‐L1 expression in this study, probably because of the short follow‐up period and the small number of enrolled patients who died. However, we confirmed that PD‐L1 expression is strongly correlated with smoking status in this study, so it might be useful for selecting patients for PD‐L1 immunotherapy in the clinic.

It was observed that PD‐L1 expression was significantly related to age and TNM stage. In recent literature, the relationship between age and PD‐L1 expression has been controversial. There are studies suggesting a positive correlation,^39^, ^40^ whereas others find no statistical correlation.^26^, ^27^ The relationship between pathological TNM stage and PD‐L1 expression has been also controversial. Some reports have the same conclusions as this study,^25^, ^37^, ^39^, ^40^ and another suggests no association.^26^, ^41^ The biology of an association between PD‐L1 expression and these clinicopathological factors is not well understood. We assume that older age and advanced TNM stage may be associated with a higher tumour‐mutation burden. Consequently, PD‐L1 expression rates were increased and PD‐L1 expression tumours showed worse prognosis.^39^, ^42^

### 4.3 Limitations

This study has a key limitation. We have no clinical outcome data on the responses to anti‐PD‐1 immunotherapy. We did not include patients prospectively treated with immunotherapeutic agents. Another limitation is the limited number of paired cases. However, it was challenging to obtain matched cytology CBs and resection specimens with sufficient tumour cells remaining in both following routine diagnosis and work‐up. We evaluated for only the concordance rate of PD‐L1 expression between cytology CBs and matched surgical resection specimens with a limited number of cases in this study. We will make every endeavour to perform further clinical concordance studies in the larger number of patients treated with PD‐1 axis therapies.

## 5 CONCLUSION

Herein, we found that cytology CB sections showed a strong positive correlation with resection specimens of the same anatomic site for PD‐L1 assessment using the SP263 IHC assay. Taken together with our findings and a few studies of PD‐L1 concordance between cytology and histology,^26^, ^27^, ^28^, ^29^, ^30^, ^31^ lung cancer patients with positive PD‐L1 expression in the preoperative or diagnostic cytology do not need to repeat the test using the surgical resection specimens, at least using the cut‐off of 1%. We propose that CBs are suitable materials for evaluating PD‐L1 expression when simultaneously performing both diagnostic and molecular tests. There is no need to perform further biopsies for other molecular tests for management of advanced‐stage lung cancer. In clinical practice, cytology specimens may represent the only specimens available, especially from patients with advanced disease. CBs prepared from residual LBC material improve the diagnostic accuracy and tumour subclassification^15^ and provide the results of a PD‐L1 test and molecular tests rapidly.

We believe that CBs prepared from residual LBC material have an important role on the application of anti‐PD‐L1 therapy and expand the pool of PD‐L1 examinations in the clinic. For therapeutic interventions, inhibition of PD‐1/PD‐L1 is expected to become a more powerful therapeutic alterative for NSCLC than ever before.

## CONFLICT OF INTEREST

The authors have no conflicts of interest to report.

## AUTHOR'S CONTRIBUTIONS

M.G.P. and M.S.R. designed and carried out the study, and drafted the manuscript. M.G.P. and M.S.R. participated in the performance of the study and performed the statistical analysis. M.G.P. concept, collection of data. M.S.R. concept, intellectual content and editing of manuscript. All authors read and approved the final manuscript.

## Data Availability

The data that support the findings of this study are available from the corresponding author upon reasonable request. The data are not publicly available due to privacy or ethical restrictions.
